# Side Effects of COVID-19 Vaccines Primer Doses: Experience of Saudi Healthcare Workers Participating in CoVaST-SA

**DOI:** 10.3390/vaccines10122137

**Published:** 2022-12-13

**Authors:** Abanoub Riad, Safa S. Alsaad, Ali A. Almurikhi, Fayez A. Alzahrani, Ali M. Alghamdi, Esra H. Alzaid, Miloslav Klugar

**Affiliations:** 1 Czech National Centre for Evidence-Based Healthcare and Knowledge Translation (Cochrane Czech Republic, Czech EBHC: JBI Centre of Excellence, Masaryk University GRADE Centre), Faculty of Medicine, Masaryk University, 625 00 Brno, Czech Republic; 2 Institute of Health Information and Statistics of the Czech Republic, 128 01 Prague, Czech Republic; 3 Department of Family Medicine, King Fahad Specialist Hospital, Dammam 32253, Saudi Arabia; 4 Department of Infectious Diseases, King Fahad Specialist Hospital, Dammam 32253, Saudi Arabia

**Keywords:** CoVaST, COVID-19 vaccines, drug-related side effects and adverse reactions, health personnel, Saudi Arabia

## Abstract

Background: Side effects emerging after COVID-19 vaccines may adversely impact public confidence in vaccines. Therefore, this study was designed to explore the short-term side effects of COVID-19 vaccines as a part of the COVID-19 Vaccines Safety Tracking (CoVaST) study. Methods: A cross-sectional survey-based study was carried out to collect data from healthcare workers (HCWs) in Saudi Arabia. The study was initiated between June and December 2021. A validated questionnaire was used in this study consisting of four categories, including demographic characteristics and medical anamnesis of the participants, COVID-19-associated anamnesis, and side effects of vaccine uptake. Results: The study included 1039 participants, of which 70.2% were females, and their median age was 34. About 82.9% and 52.3% of the participants reported a minimum of both one local and systemic side effect, respectively. Females, young participants (≤34 years old), and non-obese participants had more potential to disclose post-vaccination side effects than their counterparts. Heterologous schedules and viral vector-based vaccines were linked with a greater rate of systemic side effects, whereas homologous vaccination schedules and mRNA-based vaccines were linked with a greater rate of local side effects. Conclusion: Future studies on COVID-19 vaccines should focus on the role of BMI, previous infection, and vaccination schedule in terms of vaccine safety and reactogenicity.

## 1. Introduction

The worldwide burden related to the COVID-19 virus remains a serious public health issue [1,2]. Hence public health institutions such as the World Health Organization (WHO) regularly issue regulations to help protect people, preventing and slowing down the SARS-CoV-2 spread [3]. Moreover, the U.S. Centres for Disease Control and Prevention (CDC) have advocated various policies to reduce the spread of coronavirus disease [4]. Current evidence shows that one of the most effective measures that can reduce the risk of infection is by using vaccinations [5].

The rapid COVID-19 vaccine development and wide availability have given a ray of hope to humanity to control the ongoing global pandemic of COVID-19. However, the success of the vaccination programs depends on the population’s understanding of the risks and benefits of the vaccine and their belief in the benefit of the vaccines. Researchers found that refusal or indecision towards immunisation results from a lack of knowledge about the balance between the benefits and risks of vaccination [6,7], which is defined as vaccine hesitancy (VH) by the WHO; it refers to the “delay in acceptance or refusal of vaccines despite the availability of vaccine services” [8]. Recent studies in the Middle East revealed that COVID-19 VH was as high as 20% in Qatar and 26.2% in Kuwait [9,10]. In 2019, WHO recognised vaccine hesitancy as a major global public health threat driven by misleading and incorrect information regarding vaccines’ effectiveness and safety [11].

In September 2020, the WHO announced the introduction of several COVID-19 vaccines [12], and by the start of the year 2021, several international health regulations declared many vaccines eligible for emergency use authorisation (EUA) [13,14]. The initial vaccines authorised and launched in Saudi Arabia were Pfizer-BioNTech and Oxford-AstraZeneca vaccines. The Pfizer vaccine’s effectiveness reached 95%, and the effectiveness of the AstraZeneca vaccine was 70% [15,16]. The initial authorisation for this vaccine was given over three phases. Phase I targeted healthcare workers (HCWs) at greater risk for exposure to the virus and people over 65 years of age and older. Phase II targeted other frontline workers (healthcare workers, military and security in critical locations) and people over 50. Lastly, phase III was directed at the Saudi population, excluding children and pregnant women [17].

As a result of widely used COVID-19 vaccines, many questions and concerns were raised about obtaining COVID-19 vaccines among some people globally. For example, in Saudi Arabia, questions were raised by the general population regarding the COVID-19 vaccine programs concerning the safety of the approved vaccines. However, common side effects demonstrated by clinical trials after getting COVID-19 vaccines tend to be mild to moderate, such as fever, headache, fatigue (tiredness), redness, and pain or swelling at the site of injection [15,18]. The recent reports of some rare cases of severe side effects, such as thrombosis following COVID-19 vaccines [19], may also affect the acceptance of COVID-19 vaccines among some populations. Furthermore, there is limited data and literature reviews concerning each vaccine’s side effects. Therefore, ongoing monitoring of COVID-19 vaccines is vital to promote reassurance and acknowledgement of COVID-19 vaccines amongst the public by doing independent post-marketing studies to understand the side effects of COVID-19 vaccines better.

The primary objective of this study was to reveal the prevalence of COVID-19 vaccine-associated side effects among vaccinated healthcare workers (HCWs) by either Oxford-AstraZeneca or Pfizer BioNTech vaccines, which are currently used in Saudi Arabia. The secondary objective was to investigate the potential demographic characteristics and medical anamnesis for the intensity and frequency of side effects after taking the vaccines.

## 2. Materials and Methods

### 2.1. Design

A cross-sectional survey-based study was carried out between June and December 2021 to collect data on the side effects of the COVID-19 vaccine among recruited healthcare workers (HCWs) in all regions of Saudi Arabia. The study used a validated multiple-choice self-administered online questionnaire digitally designed using KoBoToolbox version 2.021.03 (Harvard Humanitarian Initiative, Cambridge, MA, USA, 2021) written in Arabic and English [20]. The questionnaire asked about the short-term side effects after either the first, second, or both doses of the COVID-19 vaccine. The side effects are classified as local or systemic, and their onset, duration, and intensity are self-reported. After the ethical review, an invitation to participate was sent to the target groups (HCWs) online, mainly via social media platforms (namely WhatsApp mobile applications).

### 2.2. Participants

The inclusion criteria for this study were HCWs who received the COVID-19 vaccine in the post-approval phase and those recently vaccinated who received their vaccine dose within the last 30 days were prioritised for study invitation. However, the study was not limited to recently vaccinated individuals. Eligible participants were preferably at least 18 years of age and able to provide consent independently. HCWs who received the COVID-19 vaccines as part of phase III clinical trials were excluded from the study.

### 2.3. Instrument

The standardised questionnaire focused on adverse events and side effects of COVID-19 vaccines and was previously validated in several countries. The questionnaire consisted of four categories:

i. Demographic characteristics (age, gender, height, weight, profession, and geographic region).
ii. Medical anamnesis (chronic diseases, medication, smoking and alcohol consumption).
iii. COVID-19-related medical histories (type of vaccine, number of vaccine doses, dates of vaccine doses, previous infection, and date of diagnosis).
iv. Vaccine side effects (local side effects, systemic side effects, onset, and duration).

### 2.4. Ethics

The Institutional Review Board (IRB) of King Fahad Specialist Hospital reviewed and approved the study on 1 July 2021. All investigators have also earned the Collaborative Institutional Training Initiative (CITI) certificate. The first page of the electronic survey contained a description of the study and a statement of informed consent, which was obtained from each participant prior to participation. Participants could withdraw from the study at any time without explanation, and no data was stored until the participant finally submitted their answers.

### 2.5. Analyses

The categorical variables were summarised using frequencies and percentages, while the numerical variables were summarised using means and standard deviations. The association between local and systemic side effects and their sociodemographic and anamnestic predictors was tested using the Chi-square test, Fisher’s exact test, and the Mann–Whitney test. A significance level of <0.05 (two-tailed) was used to indicate statistical significance. All data analyses were performed using Statistical Packages for Software Sciences (SPSS) Version 28.

## 3. Results

### 3.1. Demographic Characteristics

Out of the 1039 participants, 729 (70.2%) were women, and 310 (29.8%) were men. The median age of the participants was 34 years, and the most represented professions were doctors (44.3%), followed by nurses (23.4%) and pharmacists (3.4%). The mean body mass index (BMI) of the participants was 26.24 ± 5.66, which was significantly (sig. < 0.01) higher in men (27.78 ± 5.13) than in women (25.59 ± 5.75). While 42.9% were normal weight, 34.6% and 12.8% were overweight and obese, respectively. Females (47.2%) were more likely (sig. = 0.045) to be of normal weight than males (33%). Most of the participants (91.4%) came from the eastern region (Table 1).

### 3.2. Medical and COVID-19-Related Anamnesis

Less than one-fifth of participants (18.1%) reported suffering from at least one chronic disease, with a significant difference between ≤34-year-old vs. >34-year-old participants (9.8% vs. 26.4%; sig. < 0.001) and without a significant difference between females vs. males (18.5% vs. 17.1%; sig. = 0.586). Chronic hypertension was the most common condition (5.1%), followed by asthma (4.1%), thyroid disease (3.3%) and allergies (2.5%). Less than a quarter of participants (24.4%) reported taking medications regularly, with a significant difference between ≤34-year-old vs. >34-year-old participants (16% vs. 32.8%; sig. < 0.001) and without a significant difference between women vs. men (25% vs. 23.2%; sig. = 0.550). Only 8.3% reported smoking tobacco, with a significant difference between women vs. men (2.3% vs. 22.3%; sig. < 0.001) (Supplementary Table S1).

Overall, 21.1% reported being previously infected with SARS-CoV-2, most of which (66.7%) occurred before receiving the first dose, and the remainder occurred after the first (16%) and second (17.4%) doses. While 63.9% of those infected had a mild course, 34.2% and 1.8% reported moderate or severe courses. Notably, 66.6% and 50% of the moderate and severe cases were pre-vaccinated. The mean duration of infection was 9.35 ± 10.76 days with no statistically significant difference between ≤34-year-old vs. >34-year-old participants (8.54 7.35 vs. 10.14 13.51; sig. = 0.597). Most of those infected reported a mild course of the disease (63.9%), and the rest reported moderate (34.2%) and severe (1.8%) courses. The most reported symptom was fatigue (75.3%), followed by myalgia (70.8%), headache (66.2%), loss of taste/smell (63%), and fever/chills (56.2%). Nausea/vomiting was significantly (sig. = 0.006) more common in females (21.8%) than in males (6.3%) (Supplementary Table S1).

### 3.3. Post-Vaccination Side Effects by Sex, Age, and BMI

Overall, most participants (82.9%) reported at least one local adverse reaction, with statistically significant differences between women vs. men (84.6% vs. 78.7%; sig. = 0.020), ≤34-years-old vs. >34-years-old (87.5% vs. 78.2%; sig. < 0.001) and non-obese vs. obese participants (84.1% vs. 78%; sig. = 0.041).

The most frequently reported local adverse reaction was injection site pain (79.4%), followed by injection site swelling (17.6%) and injection site redness (7.7%). Women reported significantly more injection site swelling (20.7% vs. 10.3%; sig. < 0.001) and injection site redness (9.5% vs. 3.5%; sig. = 0.001) in comparison to their male peers. Participants ≤ 34 years of age reported significantly more injection site pain (84.2% vs. 74.5%; sig. < 0.001) than those older than 34. Similarly, the non-obese participants reported significantly more injection site pain than the obese participants (80.8% vs. 73.5%; sig. = 0.022) (Table 2).

More than half of the sample (52.3%) reported at least one systemic adverse reaction, with no statistically significant differences between females vs. males (53.5% vs. 49.4%; sig. = 0.221), ≤34 years old vs. >34 years old (52.9% vs. 51.5%; sig. = 0.666) and non-obese vs. obese participants (52.9% vs. 49.5%; sig. = 0.390).

The most reported systemic adverse reaction was fatigue (36.1%), followed by myalgia (26%), headache (25.2%), fever (17.8%), arthralgia (13.5%), chills (11th 1%) and nausea (7.1%). Females reported significantly more headaches (27.6% vs. 19.7%; sig. = 0.007) and nausea (8.6% vs. 3.5%; sig. = 0.003) than their male peers. Participants over 34 years of age reported significantly more lymphadenopathy (3.7% vs. 1%; sig. = 0.004) than those 34 years or less. No single systemic side effect was significantly different between non-obese and obese participants (Table 2).

Anaphylactic reactions were reported by only four participants (0.4%), and three of these (75%) were non-obese women over 34 years of age. Lymphadenopathy, oral paraesthesia, dysgeusia, oral ulcers, and rash occurred in 2.3%, 0.8%, 0.7%, 0.7%, and 0.7% of the sample. Oral and dermatological side effects did not differ significantly between gender, age, BMI, or vaccination schedule.

### 3.4. Post-Vaccination Side Effects by Dose and Vaccination Schedule

Local side effects were more common with mRNA-based vaccines than viral vector-based vaccines after the first dose (85.6% vs. 75.4%; sig. < 0.001) and the second dose (84.9% vs. 79.5%; sig. = 0.121). On the contrary, systemic side effects were more common with viral vector-based vaccines than mRNA-based vaccines after the first dose (59.6% vs. 49.5%; sig. = 0.004) and the second dose (57.6% vs. 51.8%; sig. = 0.221).

While injection site pain was significantly more common with mRNA-based vaccines than viral vector-based vaccines after the first dose (82.2% vs. 71.8%; sig. < 0.001) and the second dose (81.5% vs. 74.2%; sig. = 0.053), injection site swelling and redness were not significantly different between mRNA- and viral vector-based vaccines.

Following the first dose, fatigue (42.5% vs. 33.7%; sig. = 0.009), headache (32.5% vs. 22.5%; sig. = 0.001), myalgia (32.9% vs. 23.5%; sig. = 0.002), arthralgia (19.3% vs. 11.3%; sig. < 0.001), fever (27.5% vs. 14.2%; sig. < 0.001), and chills (16.4% vs. 9.1%; sig. < 0.001) were significantly more common after viral vector-based vaccines. Likewise, following the second dose, arthralgia (19.7% vs. 12.5%; sig. = 0.025) and fever (27.3% vs. 16.8%; sig. = 0.004) were significantly more common after viral vector-based vaccines (Table 2).

When comparing vaccination regimens, homologous schedules were significantly stronger with local side-effect incidence (85.2% vs. 77.7%; sig. = 0.025) and pain at the site of injection (81.6% vs. 74.1%; sig. = 0.041) associated as heterologous schemes. In contrast, heterologous schedules were significantly more common with headache (33.1% vs. 23.9%; sig. = 0.022), fever (26.6% vs. 16.8%; sig. = 0.006) and nausea (12.2% vs. 6.1%; sig. = 0.009) than homologous schedules (Table 2).

### 3.5. Onset and Duration of Post-Vaccination Side Effects

**Figure 1:**
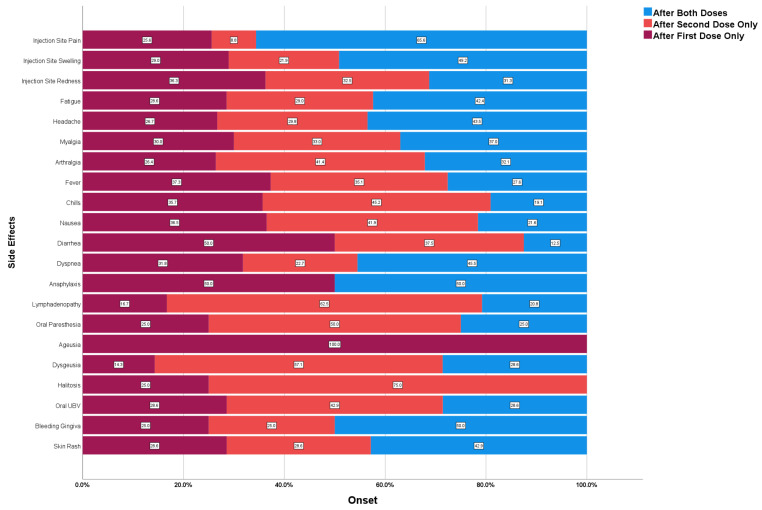
Onset of COVID-19 Side Effects Experienced by Saudi Healthcare Workers Responding to CoVaST-SA, (n = 1039).

Most participants (65.6%) reported injection site pain after both doses, while only 49.2% and 31.3% reported injection site swelling and redness after both doses. All local side effects tended to be associated with the first dose rather than the second dose, as injection site pain was reported 25.6% more often after the first dose only, compared to only 8.8% after the second dose. Dyspnoea (45.5%), headache (43.5%), fatigue (42.4%) and myalgia (37%) were the most common systemic adverse reactions reported after both doses. While diarrhoea (50%), fever (37.3%), nausea (36.5%), dyspnoea (31.8%) and myalgia (30%) were reported more frequently only after the first dose, chills (45.2%), nausea (41.9%), arthralgia (41.4%) and diarrhoea were reported more frequently after the second dose Figure 1.

**Figure 2:**
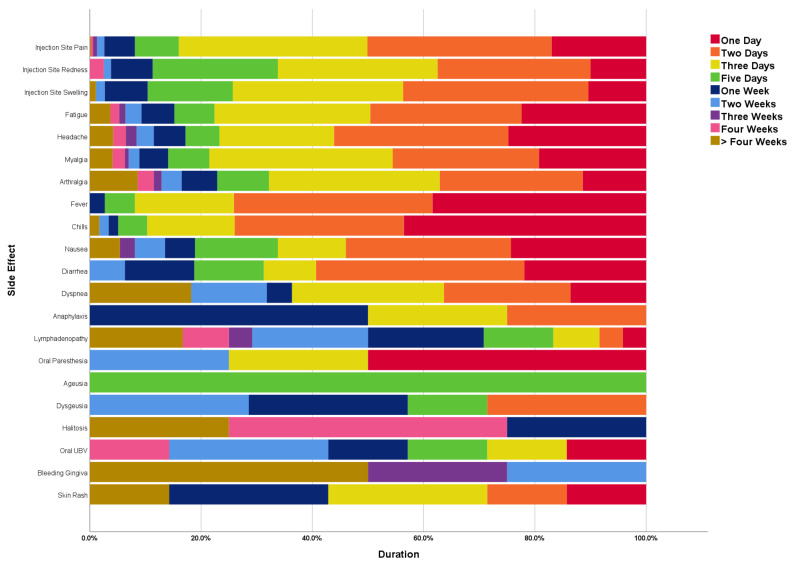
Duration of COVID-19 Side Effects Experienced by Saudi Healthcare Workers Responding to CoVaST-SA, (n = 1039).

Most local adverse reactions persisted between one and three days, including injection site pain (84%), injection site swelling (74.3%) and injection site redness (66.3%). Moreover, fatigue (77.6%), headache (76.7%), myalgia (78.5%), arthralgia (67.9%), fever (91.9%), chills (89.6%), nausea (66.2%), diarrhoea (68.8%) and dyspnoea (63.6%) persisted between one and three days. On the other hand, the lymphadenopathy persisted over longer intervals, e.g., five days (12.5%), one week (20.8%), two weeks (20.8%) and more than four weeks (16.7%). Most rashes (85.7%) resolved within the first week (Figure 2).

### 3.6. Post-Vaccination Medications

Fewer than two-thirds (62.5%) of participants reported taking post-vaccination medication to control their side effects, with acetaminophen being the most common (91.5%), followed by ibuprofen (9.2%) and diclofenac (1.5%). No statistically significant differences were found among participants in post-vaccination medication use in terms of gender, age, BMI, and medical history. Participants who received viral vector-based vaccines were significantly more inclined to stop drugs after the first (73.6% vs. 58.4%; sig. < 0.001) and second doses (78.8% vs. 60.3%; sig. < 0.001). Heterologous vaccination schedules were more significantly (72.7% vs. 61.2%; sig. = 0.010) associated with post-vaccination drug intake. Participants who reported local side effects (sig. < 0.001) or systemic side effects (sig. < 0.001) used medications more often (Table 3).

### 3.7. Risk Factors of Post-Vaccination Side Effects

When performing binary logistic regression, women were found to be 1.490 (95% CI: 1.062 2.090) times more likely to report local side effects than men. Likewise, ≤34-year-old participants (OR: 1.953; CI 95%: 1.400 2.725), non-obese (OR: 1.487; CI 95%: 1.014 2.179), participants not previously infected with SARS-CoV-2 (OR: 1.490; 95% CI: 1.028–2.159) and those receiving a homologous schedule (OR: 1.658; 95% CI: 1.062–2.588) were more likely to experience local side reactions (Table 4).

## 4. Discussion

The current study was conducted to assess the side effects experienced by Saudi healthcare workers (HCWs) after getting primer doses of COVID-19 vaccines. Up to 82.9% and 52.3% of the participants reported a minimum of one local and one systemic side effect, respectively. The most reposted local side effect was pain at the sight of infection (79.4%), while the most common systemic adverse reaction was fatigue (36.1%), followed by myalgia (26%), headache (25.2%), and fever (17.8%). Females, young participants (≤34 years old), and non-obese respondents had more potential to disclose post-vaccine side effects in comparison to their counterparts. Homologous vaccination schedules and mRNA vaccines were associated with a greater frequency of local adverse reactions, while heterologous schedules and viral vector-based preparations were associated with a greater frequency of systemic side reactions.

In our study, females reported more local (84.6% vs. 78.7%) and systemic (53.5% vs. 49.4%) side effects than males, which is consistent with what had been previously reported among Saudi and non-Saudi populations. El-Shitany et al., 2021 discussed a cross-sectional study of the immediate adverse effects experienced by Saudi residents after receiving an mRNA-based vaccine (BNT162b2) and found that females (58%) were significantly more likely to report post-vaccine side effects than males (28.1%) [21]. Females’ susceptibility was evident in several studies conducted in Saudi Arabia, such as that by Ahsan et al., 2021, who found that female HCWs were more likely to report side effects (93.1% vs. 57.4%; sig. < 0.001) than males [22]. Likewise, Darraj et al., 2022 reported Saudi female HCWs’ susceptibility after receiving a viral vector-based vaccine (ChAdOx1-S) [23]. Additionally, Mohammed et al., 2021 [24], Alghamdi et al., 2021 [25], and Alzarea et al., 2022 [26] found a phenomenon of females’ predisposition among the general Saudi population. Among children aged between 12 and 18, female participants reported more side effects after receiving an mRNA-based vaccine (BNT162b2) than their male counterparts in Saudi Arabia [27].

Moreover, non-Saudi studies had confirmed females’ predisposition, e.g., studies among Czech HCWs after BNT162b2 [28], Slovak HCWs after mRNA-based vaccines [29], German HCWs after mRNA- and viral vector-based vaccines [30], Ethiopian HCWs after ChAdOx1-S [31], Turkish HCWs after CoronaVac [32], and Algerian and Polish HCWs after receiving primer doses of COVID-19 vaccines [33,34].

Green et al., 2022 analysed four cross-sectional studies that were carried out after disseminating two and three doses of BNT162b2, and they found females’ predisposition consistently among all age groups [35]. One of the proposed explanations for this phenomenon is that females may have a more enhanced immune response which can be evident through their lower COVID-19 case-fatality rates [36]. Moreover, females exhibit higher levels of type IFN I and innate immune responses, which are believed to be subjected to modulation by X chromosome-linked and ChrY gene polymorphisms [37,38,39].

Our young participants (≤34 years old) experienced more local (87.5% vs. 78.2%) and systemic (52.9% vs. 51.5%) side effects than older participants (>34 years old), which is consistent with what was reported among Saudi and non-Saudi populations. According to Ahsan et al., 2021, younger Saudi adults (≤36 years old) experienced more side effects than their older counterparts (>36 years old) after receiving BNT162b2 and ChAdOx1-S [22]. Likewise, Alzarea et al., 2022 revealed that younger Saudi adults (≤35 years old) experienced more side effects than their older counterparts (>35 years old) after receiving BNT162b2 and ChAdOx1-S [26]. A recent systematic review of clinical trials found that younger adults were more likely to have neurological and muscular adverse effects, such as headache and myalgia, than their older counterparts after receiving COVID-19 vaccines [40]. The age-related differences in side-effect incidence can be explained by the varying levels of binding antibodies, which were reportedly lower among seniors and older adults [41,42].

The lower BMI levels were associated with higher frequencies of local (84.1% vs. 78%) and systemic (52.9% vs. 49.5%) side effects among our participants. Likewise, a Spanish cross-sectional study found that non-obese status was significantly associated with a greater frequency of post-vaccine side effects [43]. In Iran, headache was significantly more common among non-obese individuals, while flu-like symptoms were more common among the obese ones [44]. Pellini et al., 2021 evaluated levels of antibody titers after BNT162b2 among HCWs and found that non-obese participants had a more efficient humoral response compared with obese participants [45].

While mRNA-based vaccines recipients reported more local side effects after the first (85.6% vs. 75.4%) and second doses (84.9% vs. 79.5%), viral vector-based vaccines recipients reported more systemic side effects after the first (59.6% vs. 49.5%) and second doses (57.6% vs. 51.8%). Similarly, Klugar et al., 2021 revealed that local reactions were more associated with mRNA vaccines, while systemic adverse effects were associated with viral vector-based vaccines among German HCWs [30]. In Poland, Andrzejczak-Grządko et al., 2021 indicated that BNT162b2 was associated with more frequent local adverse effects, i.e., injection site pain and arm pain and less frequent systemic side effects (headache, myalgia, headache, fever, and chills) [46].

The homologous schedules were associated with a greater frequency of local adverse effects and a lower frequency of systemic side effects compared with heterologous schedules in our study. Rzymski et al., 2022 revealed that homologous schedules of mRNA-based vaccines had significantly higher levels of post-vaccination side effects [47]. In Brazil, heterologous vaccination schedules produced more robust immune responses than homologous schedules, and they were associated with more side effects [48]. Likewise, Schmidt et al., 2021 found that local and systemic adverse effects were less common in post-homologous schedules (vector-vector) than in heterologous schedules (mRNA-vector) among German adults [49].

Fewer than two-thirds (62.5%) of our participants reported using medications after vaccination to control their side effects, with acetaminophen being the most common (91.5%), followed by ibuprofen (9.2%), and diclofenac (1.5%). However, there is a paucity of evidence about the interference of analgesics/antipyretics with the vaccine-elicited immune response; it is unlikely that these medications might have any significant impact on vaccine effectiveness [50]. Moreover, there is a lack of evidence about the benefits of these medications against serious adverse reactions, even though public health authorities recommend using these medications to control post-vaccination side effects [50]. Iguacel et al., 2021 found that 62.7% of Spanish vaccinees reported using analgesics such as acetaminophen and ibuprofen to control/relieve their side effects [43]. The same finding was reported in Nepal [51] and Ghana [52].

### 4.1. Strengths

This study used a validated instrument (questionnaire) that had been broadly used by a series of cross-sectional studies in multiple countries, thus facilitating comparisons. The identity of participants was kept anonymous to reduce information bias. The present study attempted to evaluate the role of various demographic and anamnestic risk factors in terms of post-vaccine side effects emergence, onset, and duration. This study employed HCWs as the target population because they supposedly retain the highest levels of health literacy within their respective communities.

### 4.2. Limitations

Firstly, the cross-sectional design deprived us of following the adverse reactions that might remain longer than the standard period. The number of participants was not equally or proportionately distributed among geographic regions, age groups, or sex. Given the current sample size, very rare side effects cannot be captured or validated.

### 4.3. Implications

Future studies on COVID-19 vaccines should focus on the role of BMI and previous infection in vaccine safety and reactogenicity. The differences between homologous and heterologous vaccination schedules should be further evaluated through various scenarios. The older participants had fewer side effects; possibly implying that they could be protected through prioritisation schemes of booster doses.

## 5. Conclusions

The present study found that 82.9% and 52.3% of the participants reported a minimum of one local and systemic side effect, respectively. Females, young participants (≤34 years old), and non-obese participants had more potential to disclose post-vaccine side effects than their counterparts. Future studies on COVID-19 vaccines should focus on the role of BMI, previous infection, and vaccination schedule in terms of vaccine safety and reactogenicity.

## Figures and Tables

**Table 1:** Demographic Characteristics of Saudi Healthcare Workers Responding to CoVaST-SA, (*n* = 1039).

Variable	Outcome	Female (*n* = 729)	Male (*n* = 310)	Total (*n* = 1039)	*Sig.*
Age	≤34 years old	387 (53.2%)	133 (42.9%)	520 (50.1%)	**<0.002**
	>34 years old	341 (46.8%)	177 (57.1%)	518 (49.9%)	
	µ ± SD	36.25 ± 18.64	36.99 ± 8.86	36.47 ± 16.34	**0.017**
Profession	M.D.	309 (42.4%)	151 (48.7%)	460 (44.3%)	0.060
	D.D.S.	15 (2.1%)	9 (2.9%)	24 (2.3%)	0.406
	R.N.	200 (27.4%)	43 (13.9%)	243 (23.4%)	**<0.001**
	Pharmacist	18 (2.5%)	17 (5.5%)	35 (3.4%)	**0.014**
	Physiotherapist	6 (0.8%)	2 (0.6%)	8 (0.8%)	1.000 *
	Other	181 (24.8%)	88 (28.4%)	269 (25.9%)	0.231
BMI	Underweight (<18.5)	32 (4.4%)	0 (0%)	32 (3.1%)	**<0.001**
	Normal (18.5–24.9)	342 (47.2%)	102 (33%)	444 (42.9%)	**<0.001**
	Overweight (25–29.9)	237 (32.7%)	121 (39.2%)	358 (34.6%)	**0.045**
	Obese (30–34.9)	74 (10.2%)	58 (18.8%)	132 (12.8%)	**<0.001**
	Extremely Obese (>35)	40 (5.5%)	28 (9.1%)	68 (6.6%)	**0.035**
	µ ± SD	25.59 ± 5.75	27.78 ± 5.13	26.24 ± 5.66	**<0.001**
Region	Eastern	670 (91.9%)	280 (90.3%)	950 (91.4%)	0.404
	Central	38 (5.2%)	23 (7.4%)	61 (5.9%)	0.166
	Western	15 (2.1%)	4 (1.3%)	19 (1.8%)	0.461
	Northern	2 (0.3%)	1 (0.3%)	3 (0.3%)	1.000 *
	Southern	4 (0.5%)	2 (0.6%)	6 (0.6%)	1.000 *

Chi-squared (*χ^2^*), Fisher’s exact (*) and Mann–Whitney (*U*) tests were used with a significance level (*sig.*) < 0.05. The significant values are in bold font.

**Table 2:** Prevalence of COVID-19 Side Effects Experienced by Saudi Healthcare Workers Responding to CoVaST-SA, (*n* = 1039).

Variable	Outcome	Sex		*Sig.*	Age		*Sig.*	BMI		*Sig.*	
		Female (*n* = 729)	Male (*n* = 310)		≤34 years (*n* = 520)	>34 years (*n* = 518)		Non-Obese (*n* = 834)	Obese (*n* = 200)		
Local SE	Injection Site Pain	589 (80.8%)	236 (76.1%)	0.089	438 (84.2%)	386 (74.5%)	**<0.001**	674 (80.8%)	147 (73.5%)	**0.022**	
	Injection Site Swelling	151 (20.7%)	32 (10.3%)	**<0.001**	87 (16.7%)	96 (18.5%)	0.446	145 (17.4%)	38 (19%)	0.591	
	Injection Site Redness	69 (9.5%)	11 (3.5%)	**0.001**	35 (6.7%)	45 (8.7%)	0.237	59 (7.1%)	21 (10.5%)	0.103	
	Total	617 (84.6%)	244 (78.7%)	**0.020**	455 (87.5%)	405 (78.2%)	**<0.001**	701 (84.1%)	156 (78%)	**0.041**	
Systemic SE	Fatigue	261 (35.8%)	114 (36.8%)	0.765	189 (36.3%)	185 (35.7%)	0.832	303 (36.3%)	71 (35.5%)	0.826	
	Headache	201 (27.6%)	61 (19.7%)	**0.007**	139 (26.7%)	123 (23.7%)	0.268	218 (26.1%)	43 (21.5%)	0.175	
	Myalgia	191 (26.2%)	79 (25.5%)	0.810	135 (26%)	135 (26.1%)	0.971	225 (27%)	42 (21%)	0.083	
	Arthralgia	102 (14%)	38 (12.3%)	0.454	69 (13.3%)	71 (13.7%)	0.837	118 (14.1%)	22 (11%)	0.242	
	Fever	130 (17.8%)	55 (17.7%)	0.972	98 (18.8%)	87 (16.8%)	0.388	151 (18.1%)	32 (16%)	0.483	
	Chills	83 (11.4%)	32 (10.3%)	0.617	65 (12.5%)	50 (9.7%)	0.144	98 (11.8%)	17 (8.5%)	0.189	
	Nausea	63 (8.6%)	11 (3.5%)	**0.003**	37 (7.1%)	37 (7.1%)	0.986	62 (7.4%)	12 (6%)	0.480	
	Diarrhoea	25 (3.4%)	7 (2.3%)	0.317	12 (2.3%)	20 (3.9%)	0.148	25 (3%)	7 (3.5%)	0.713	
	Dyspnea	18 (2.5%)	4 (1.3%)	0.227	10 (1.9%)	12 (2.3%)	0.660	19 (2.3%)	3 (1.5%)	0.784	
	Anaphylaxis	3 (0.4%)	1 (0.3%)	1.000 *	1 (0.2%)	3 (0.6%)	0.373 *	4 (0.5%)	0 (0%)	1.000 *	
	Lymphadenopathy	21 (2.9%)	3 (1%)	0.060	5 (1%)	19 (3.7%)	**0.004**	20 (2.4%)	4 (2%)	1.000 *	
	Oral Paresthesia	8 (1.1%)	0 (0%)	0.114 *	4 (0.8%)	4 (0.8%)	1.000 *	6 (0.7%)	2 (1%)	0.656 *	
	Ageusia	1 (0.1%)	0 (0%)	1.000 *	0 (0%)	1 (0.2%)	0.499 *	1 (0.1%)	0 (0%)	1.000 *	
	Dysgeusia	7 (1%)	0 (0%)	0.111 *	3 (0.6%)	4 (0.8%)	0.725 *	6 (0.7%)	1 (0.5%)	1.000 *	
	Halitosis	3 (0.4%)	1 (0.3%)	1.000 *	1 (0.2%)	3 (0.6%)	0.373 *	4 (0.5%)	0 (0%)	1.000 *	
	Oral UBV	6 (0.8%)	1 (0.3%)	0.681 *	3 (0.6%)	4 (0.8%)	0.725 *	4 (0.5%)	3 (1.5%)	0.136 *	
	Bleeding Gingiva	4 (0.5%)	0 (0%)	0.324 *	1 (0.2%)	3 (0.6%)	0.373 *	4 (0.5%)	0 (0%)	1.000 *	
	Skin Rash	6 (0.8%)	1 (0.3%)	0.681 *	6 (1.2%)	1 (0.2%)	0.124 *	7 (0.8%)	0 (0%)	0.357 *	
	Total	390 (53.5%)	153 (49.4%)	0.221	275 (52.9%)	267 (51.5%)	0.666	441 (52.9%)	99 (49.5%)	0.390	
**Variable**	**Outcome**	**First Dose**		***Sig.***	**Second Dose**		***Sig.***	**Schedule**		***Sig.***	**Total** **(*n* = 1039)**
		**mRNA** **(*n* = 759)**	**Vector** **(*n* = 280)**		**mRNA** **(*n* = 793)**	**Vector** **(*n* = 132)**		**Homologous** **(*n* = 786)**	**Heterologous** **(*n* = 139)**		
Local SE	Injection Site Pain	624 (82.2%)	201 (71.8%)	**<0.001**	646 (81.5%)	98 (74.2%)	**0.053**	641 (81.6%)	103 (74.1%)	**0.041**	825 (79.4%)
	Injection Site Swelling	141 (18.6%)	42 (15%)	0.179	140 (17.7%)	25 (18.9%)	0.721	148 (18.8%)	17 (12.2%)	0.061	183 (17.6%)
	Injection Site Redness	59 (7.8%)	21 (7.5%)	0.883	58 (7.3%)	14 (10.6%)	0.191	63 (8%)	9 (6.5%)	0.532	80 (7.7%)
	Total	650 (85.6%)	211 (75.4%)	**<0.001**	673 (84.9%)	105 (79.5%)	0.121	670 (85.2%)	108 (77.7%)	**0.025**	861 (82.9%)
Systemic SE	Fatigue	256 (33.7%)	119 (42.5%)	**0.009**	285 (35.9%)	54 (40.9%)	0.273	288 (36.6%)	51 (36.7%)	0.991	375 (36.1%)
	Headache	171 (22.5%)	91 (32.5%)	**0.001**	193 (24.3%)	41 (31.1%)	0.100	188 (23.9%)	46 (33.1%)	**0.022**	262 (25.2%)
	Myalgia	178 (23.5%)	92 (32.9%)	**0.002**	203 (25.6%)	40 (30.3%)	0.256	199 (25.3%)	44 (31.7%)	0.118	270 (26%)
	Arthralgia	86 (11.3%)	54 (19.3%)	**<0.001**	99 (12.5%)	26 (19.7%)	**0.025**	101 (12.8%)	24 (17.3%)	0.160	140 (13.5%)
	Fever	108 (14.2%)	77 (27.5%)	**<0.001**	133 (16.8%)	36 (27.3%)	**0.004**	132 (16.8%)	37 (26.6%)	**0.006**	185 (17.8%)
	Chills	69 (9.1%)	46 (16.4%)	**<0.001**	82 (10.3%)	20 (15.2%)	0.102	83 (10.6%)	19 (13.7%)	0.281	115 (11.1%)
	Nausea	48 (6.3%)	26 (9.3%)	0.100	52 (6.6%)	13 (9.8%)	0.171	48 (6.1%)	17 (12.2%)	**0.009**	74 (7.1%)
	Diarrhoea	20 (2.6%)	12 (4.3%)	0.172	21 (2.6%)	8 (6.1%)	0.054 *	23 (2.9%)	6 (4.3%)	0.424 *	32 (3.1%)
	Dyspnea	13 (1.7%)	9 (3.2%)	0.136	14 (1.8%)	5 (3.8%)	0.173 *	16 (2%)	3 (2.2%)	1.000 *	22 (2.1%)
	Anaphylaxis	4 (0.5%)	0 (0%)	0.579 *	3 (0.4%)	0 (0%)	1.000 *	3 (0.4%)	0 (0%)	1.000 *	4 (0.4%)
	Lymphadenopathy	19 (2.5%)	5 (1.8%)	0.494	20 (2.5%)	1 (0.8%)	0.342 *	18 (2.3%)	3 (2.2%)	1.000 *	24 (2.3%)
	Oral Paresthesia	7 (0.9%)	1 (0.4%)	0.690 *	8 (1%)	0 (0%)	0.610 *	7 (0.9%)	1 (0.7%)	1.000 *	8 (0.8%)
	Ageusia	0 (0%)	1 (0.4%)	0.269 *	1 (0.1%)	0 (0%)	1.000 *	0 (0%)	1 (0.7%)	0.150 *	1 (0.1%)
	Dysgeusia	6 (0.8%)	1 (0.4%)	0.682 *	6 (0.8%)	1 (0.8%)	1.000 *	7 (0.9%)	0 (0%)	0.603 *	7 (0.7%)
	Halitosis	2 (0.3%)	2 (0.7%)	0.295 *	2 (0.3%)	1 (0.8%)	0.370 *	2 (0.3%)	1 (0.7%)	0.387 *	4 (0.4%)
	Oral UBV	5 (0.7%)	2 (0.7%)	1.000 *	4 (0.5%)	1 (0.8%)	0.538 *	4 (0.5%)	1 (0.7%)	0.558 *	7 (0.7%)
	Bleeding Gingiva	3 (0.4%)	1 (0.4%)	1.000 *	2 (0.3%)	1 (0.8%)	0.370 *	3 (0.4%)	0 (0%)	1.000 *	4 (0.4%)
	Skin Rash	6 (0.8%)	1 (0.4%)	0.682 *	4 (0.5%)	1 (0.8%)	0.538 *	5 (0.6%)	0 (0%)	1.000 *	7 (0.7%)
	Total	376 (49.5%)	167 (59.6%)	**0.004**	411 (51.8%)	76 (57.6%)	0.221	408 (51.9%)	79 (56.8%)	0.284	543 (52.3%)

Chi-squared (*χ^2^*) and Fisher’s exact (*) test were used with a significance level (*sig.*) < 0.05. The significant values are in bold font.

**Table 3:** Prevalence of Medications Consumption Following COVID-19 Vaccination Reported by Saudi Healthcare Workers Responding to CoVaST-SA, (*n* = 1039).

Variable	Outcome	Acetaminophen (*n* = 594)	Ibuprofen (*n* = 60)	Diclofenac (*n* = 10)	Total (*n* = 649)	*Sig.*
Sex	Female	430 (59%)	43 (5.9%)	8 (1.1%)	468 (64.2%)	0.077
	Male	164 (52.9%)	17 (5.5%)	2 (0.6%)	181 (58.4%)	
Age Group	≤34 years old	304 (58.5%)	26 (5%)	6 (1.2%)	331 (63.7%)	0.451
	>34 years old	290 (56%)	34 (6.6%)	4 (0.8%)	318 (61.4%)	
BMI	Non-obese	485 (58.2%)	45 (5.4%)	10 (1.2%)	532 (63.8%)	0.056
	Obese	106 (53%)	14 (7%)	0 (0%)	113 (56.5%)	
Chronic Illnesses	Yes	116 (61.7%)	10 (5.3%)	1 (0.5%)	123 (65.4%)	0.354
	No	478 (56.2%)	50 (5.9%)	9 (1.1%)	526 (61.8%)	
Medications	Yes	152 (59.8%)	13 (5.1%)	3 (1.2%)	166 (65.4%)	0.274
	No	442 (56.3%)	47 (6%)	7 (0.9%)	483 (61.5%)	
First Dose	mRNA	402 (53%)	35 (4.6%)	7 (0.9%)	443 (58.4%)	**<0.001**
	Viral Vector	192 (68.6%)	25 (8.9%)	3 (1.1%)	206 (73.6%)	
Second Dose	mRNA	443 (55.9%)	41 (5.2%)	7 (0.9%)	478 (60.3%)	**<0.001**
	Viral Vector	93 (70.5%)	17 (12.9%)	1 (0.8%)	104 (78.8%)	
Schedule	Homologous	439 (55.9%)	49 (6.2%)	7 (0.9%)	481 (61.2%)	**0.010**
	Heterologous	97 (69.8%)	9 (6.5%)	1 (0.7%)	101 (72.7%)	
Local SE	Injection Site Pain	489 (59.3%)	53 (6.4%)	8 (1%)	538 (65.2%)	**<0.001**
	Injection Site Swelling	118 (64.5%)	15 (8.2%)	2 (1.1%)	134 (73.2%)	**<0.001**
	Injection Site Redness	46 (57.5%)	8 (10%)	2 (2.5%)	53 (66.3%)	0.467
	Total	508 (59%)	57 (6.6%)	8 (0.9%)	561 (65.2%)	**<0.001**
Systemic SE	Fatigue	282 (75.2%)	33 (8.8%)	4 (1.1%)	306 (81.6%)	**<0.001**
	Headache	211 (80.5%)	26 (9.9%)	4 (1.5%)	227 (86.6%)	**<0.001**
	Myalgia	203 (75.2%)	28 (10.4%)	3 (1.1%)	221 (81.9%)	**<0.001**
	Arthralgia	104 (74.3%)	14 (10%)	3 (2.1%)	118 (84.3%)	**<0.001**
	Fever	157 (84.9%)	22 (11.9%)	1 (0.5%)	168 (90.8%)	**<0.001**
	Chills	100 (87%)	15 (13%)	1 (0.9%)	104 (90.4%)	**<0.001**
	Nausea	53 (71.6%)	5 (6.8%)	0 (0%)	59 (79.7%)	**0.001**
	Diarrhea	23 (71.9%)	3 (9.4%)	1 (3.1%)	27 (84.4%)	**0.009**
	Dyspnea	15 (68.2%)	0 (0%)	0 (0%)	17 (77.3%)	0.147
	Anaphylaxis	3 (75%)	0 (0%)	0 (0%)	3 (75%)	1.000 *
	Lymphadenopathy	12 (50%)	3 (12.5%)	0 (0%)	17 (70.8%)	0.392
	Oral Paresthesia	7 (87.5%)	1 (12.5%)	0 (0%)	7 (87.5%)	0.271 *
	Ageusia	1 (100%)	0 (0%)	0 (0%)	1 (100%)	1.000 *
	Dysgeusia	7 (100%)	1 (14.3%)	0 (0%)	7 (100%)	**0.050** *
	Halitosis	3 (75%)	0 (0%)	0 (0%)	3 (75%)	1.000 *
	Oral UBV	4 (57.1%)	1 (14.3%)	0 (0%)	5 (71.4%)	0.717 *
	Bleeding Gingiva	1 (25%)	0 (0%)	0 (0%)	2 (50%)	0.634 *
	Skin Rash	4 (57.1%)	0 (0%)	0 (0%)	5 (71.4%)	0.717 *
	Total	379 (69.8%)	45 (8.3%)	6 (1.1%)	419 (77.2%)	**<0.001**

Chi-squared (*χ^2^*) and Fisher’s exact (*) test were used with a significance level (*sig.*) < 0.05. The significant values are in bold font.

**Table 4:** Risk Factors of COVID-19 Vaccines Side Effects Experienced by Saudi Healthcare Workers Responding to CoVaST-SA, (*n* = 1039).

Predictor	Local Side Effects			Systemic Side Effects		
	B (SE)	OR (CI 95%)	*Sig.*	B (SE)	OR (CI 95%)	*Sig.*
Sex: Female [*n* = 729] (*vs*. Male [*n* = 310])	0.399 (0.173)	1.490 (1.062–2.090)	**0.021**	0.166 (0.136)	1.181 (0.905–1.540)	0.221
Age Group: ≤34 [*n* = 520] (vs. >34 y [*n* = 518])	0.669 (0.170)	1.953 (1.400–2.725)	**<0.001**	0.054 (0.124)	1.055 (0.827 –1.346)	0.666
BMI: non-obese [*n* = 834] (vs. obese [*n* = 200])	0.396 (0.195)	1.487 (1.014–2.179)	**0.042**	0.135 (0.158)	1.145 (0.841–1.559)	0.391
Infection: No [*n* = 820] (vs. Yes [*n* = 219])	0.399 (0.189)	1.490 (1.028–2.159)	**0.035**	0.265 (0.152)	1.304 (0.967–1.758)	0.082
Schedule: Homo- [*n* = 786] (vs. Heterologous [*n* = 139])	0.506 (0.227)	1.658 (1.062–2.588)	0.026	−0.199 (0.186)	0.820 (0.570–1.179)	0.284

The significant values are in bold font.

## Data Availability

The data that support the findings of this study are available from the corresponding author upon reasonable request.

## Supplementary Materials

The following supporting information can be downloaded at: https://www.mdpi.com/article/10.3390/vaccines10122137/s1, Table S1. Anamnestic Characteristics of Saudi Healthcare Workers Responding to CoVaST-SA.
