# Supplementary material for: Side Effects of COVID-19 Vaccines Primer Doses: Experience of Saudi Healthcare Workers Participating in CoVaST-SA
Source: Vaccines (Basel). 2022 Dec 13;10(12):2137. doi: 10.3390/vaccines10122137 (PMC9788140; doi:10.3390/vaccines10122137)
Supplement: Supplementary file 1 [file vaccines-10-02137-s001.zip › vaccines-2030885-supplementary.pdf]

Table S1. Anamnestic Characteristics of Saudi Healthcare Workers Responding to CoVaST-SA, (n = 1039) 1

Variable	Outcome	Female (n = 729)	Male (n = 310)	Total (n = 1039)	*Sig.*
Chronic Illnesses	Allergy	22 (3%)	4 (1.3%)	26 (2.5%)	0.103
	Asthma	31 (4.3%)	12 (3.9%)	43 (4.1%)	0.778
	Blood Disease	9 (1.2%)	0 (0%)	9 (0.9%)	0.065 *
	Bowel Disease	3 (0.4%)	0 (0%)	3 (0.3%)	0.559 *
	Cancer	2 (0.3%)	0 (0%)	2 (0.2%)	1.000 *
	Cardiac Disease	1 (0.1%)	2 (0.6%)	3 (0.3%)	0.214 *
	Chronic Hypertension	31 (4.3%)	22 (7.1%)	53 (5.1%)	0.057
	Diabetes Mellitus I	9 (1.2%)	4 (1.3%)	13 (1.3%)	1.000 *
	Diabetes Mellitus II	12 (1.6%)	9 (2.9%)	21 (2%)	0.188
	Hepatologic Disease	2 (0.3%)	1 (0.3%)	3 (0.3%)	1.000 *
	Psychologic Distress	7 (1%)	1 (0.3%)	8 (0.8%)	0.448 *
	Neurologic Disease	8 (1.1%)	0 (0%)	8 (0.8%)	0.114 *
	Ophthalmologic Disease	1 (0.1%)	0 (0%)	1 (0.1%)	1.000 *
	Renal Disease	2 (0.3%)	2 (0.6%)	4 (0.4%)	0.587 *
	Rheumatoid Arthritis	9 (1.2%)	0 (0%)	9 (0.9%)	0.065 *
	Thyroid Disease	32 (4.4%)	2 (0.6%)	34 (3.3%)	**0.002**
	Other	31 (4.3%)	11 (3.5%)	42 (4%)	0.598
	Total	135 (18.5%)	53 (17.1%)	188 (18.1%)	0.586
Medications	Anti-asthmatic	18 (2.5%)	9 (2.9%)	27 (2.6%)	0.687
	Antibiotics	6 (0.8%)	2 (0.6%)	8 (0.8%)	1.000 *
	Anticoagulants	5 (0.7%)	2 (0.6%)	7 (0.7%)	1.000 *
	Antidepressants	15 (2.1%)	9 (2.9%)	24 (2.3%)	0.406
	Antidiabetics	20 (2.7%)	11 (3.5%)	31 (3%)	0.485
	Antiepileptics	5 (0.7%)	0 (0%)	5 (0.5%)	0.330 *
	Antihistamine	18 (2.5%)	4 (1.3%)	22 (2.1%)	0.227
	Antihypertensive	37 (5.1%)	24 (7.7%)	61 (5.9%)	0.094
	Anti-reflux	21 (2.9%)	11 (3.5%)	32 (3.1%)	0.569
	Immunosuppressive	9 (1.2%)	0 (0%)	9 (0.9%)	0.065 *
	Cholesterol Lowering	14 (1.9%)	18 (5.8%)	32 (3.1%)	**< 0.001**
	Common Analgesics	19 (2.6%)	3 (1%)	22 (2.1%)	0.093
	Contraceptives	14 (1.9%)	0 (0%)	14 (1.3%)	**0.014 ***
	Corticosteroids	3 (0.4%)	1 (0.3%)	4 (0.4%)	1.000 *
	NSAID	14 (1.9%)	3 (1%)	17 (1.6%)	0.268
	Opioid Analgesics	1 (0.1%)	0 (0%)	1 (0.1%)	1.000 *
	Thyroid Hormones	35 (4.8%)	4 (1.3%)	39 (3.8%)	**0.006**
	Other	37 (5.1%)	8 (2.6%)	45 (4.3%)	0.071
	Total	182 (25%)	72 (23.2%)	254 (24.4%)	0.550
Tobacco Smoking	Yes	17 (2.3%)	69 (22.3%)	86 (8.3%)	**< 0.001**

	No	712 (97.7%)	241 (77.7%)	953 (91.7%)	
	Daily Cigarettes (μ ± SD)	6.24 ± 5.75	14.06 ± 8.99	12.51 ± 8.99	**0.001**
COVID-19 Infection	Yes †	156 (21.4%)	63 (20.3%)	219 (21.1%)	0.697
	No	573 (78.6%)	247 (79.7%)	820 (78.9%)	
† Infection Onset	Before First Dose	107 (68.6%)	39 (61.9%)	146 (66.7%)	0.342
	After First Dose	21 (13.5%)	14 (22.2%)	35 (16%)	0.109
	After Second Dose	28 (17.9%)	10 (15.9%)	38 (17.4%)	0.713
† Infection Duration	μ ± SD	9.24 ± 11.17	9.60 ± 9.73	9.35 ± 10.76	0.992
† Infection Severity	Mild	103 (66%)	37 (58.7%)	140 (63.9%)	0.309
	Moderate	50 (32.1%)	25 (39.7%)	75 (34.2%)	0.281
	Severe	3 (1.9%)	1 (1.6%)	4 (1.8%)	1.000 *
† Infection Signs & Symptoms	Fever / Chills	82 (52.8%)	41 (65.1%)	123 (56.2%)	0.091
	Cough	66 (42.3%)	34 (54%)	100 (45.7%)	0.117
	Dyspnea	42 (26.9%)	13 (20.6%)	55 (25.1%)	0.331
	Fatigue	113 (72.4%)	52 (82.5%)	165 (75.3%)	0.116
	Myalgia	113 (72.4%)	42 (66.7%)	155 (70.8%)	0.395
	Headache	100 (64.1%)	45 (71.4%)	145 (66.2%)	0.299
	Smell / Taste Loss	99 (63.5%)	39 (61.9%)	138 (63%)	0.829
	Sore Throat	62 (39.7%)	19 (30.2%)	81 (37%)	0.184
	Runny Nose	79 (50.6%)	27 (42.9%)	106 (48.4%)	0.297
	Nausea / Vomiting	34 (21.8%)	5 (6.3%)	38 (17.4%)	**0.006**
	Diarrhoea	37 (23.7%)	10 (15.9%)	47 (21.5%)	0.201
	Other	22 (14.1%)	3 (4.8%)	25 (11.4%)	**0.049**

Chi-squared (χ^2), Fisher's exact (*) and Mann-Whitney (*U*) tests were used with a significance level (*Sig.*) < 0.05
